# Does Delayed Restoration Improve Shear Bond Strength of Different Restorative Protocols to Calcium Silicate-Based Cements?

**DOI:** 10.3390/ma11112216

**Published:** 2018-11-08

**Authors:** Paulo J. Palma, Joana A. Marques, Rui I. Falacho, Alexandra Vinagre, João Miguel Santos, João Carlos Ramos

**Affiliations:** Department of Dentistry, Faculty of Medicine, University of Coimbra, 3000-075 Coimbra, Portugal; ppalma@uc.pt (P.J.P.); joanaamarques@hotmail.com (J.A.M.); rifalacho@fmed.uc.pt (R.I.F.); a_vinagre@yahoo.com (A.V.); jcramos@fmed.uc.pt (J.C.R.)

**Keywords:** Biodentine, calcium silicate-based cements, mineral trioxide aggregate, regenerative endodontic procedures, shear bond strength, universal bonding agent

## Abstract

The purpose of the present study was to assess the proper time to perform a restoration (immediately or delayed) after placement of two calcium silicate-based cements (CSCs) and to test the performance of two different restorative protocols regarding shear bond strength (SBS). Seventy-five acrylic blocks were randomly divided into five groups (*n* = 15). Specimens were filled with either ProRoot MTA (Dentsply Tulsa Dental) or Biodentine (Septodont). The restoration was performed at an immediate (12 min) or delayed (seven days) timeframe, using a resin-based flowable composite (SDR) (bonded to the CSC using a universal bonding system) or glass ionomer cement (GIC) as restorative materials. SBS was measured using a universal testing machine. Fractured surfaces were evaluated, and the pattern was registered. Statistical analysis was performed using the Dunn–Sidak post hoc test (*P* < 0.05). Biodentine/immediate SDR showed the highest mean SBS value (4.44 MPa), with statistically significant differences when compared to mineral trioxide aggregate (MTA)/GIC (1.14 MPa) and MTA/immediate SDR (1.33 MPa). MTA/GIC and MTA/immediate SDR did not present significant differences regarding SBS. No statistical differences were verified concerning mean SBS between both CSCs within the 7 day groups. MTA/delayed SDR (3.86 MPa) presented statistical differences compared to MTA/immediate SDR, whereas no differences were observed regarding Biodentine performance (Biodentine/immediate SDR and Biodentine/delayed SDR (3.09 MPa)). Bonding procedures directly on top of MTA might be preferably performed at a delayed timeframe, whereas Biodentine might allow for immediate restoration.

## 1. Introduction

Tooth decay, restorative procedures, and several traumatic injuries may lead to pulp exposure and jeopardize both vitality and a successful treatment prognosis [1,2]. In some clinical situations, vital pulp therapy involves directly placing a biomaterial over the exposed pulp site (direct pulp capping) with the ultimate goal of maintaining pulp vitality by protecting the dentin-pulp complex [1,2,3,4,5]. Furthermore, vital pulp therapy approaches include indirect pulp capping (biocompatible materials used as a protective barrier) and pulpotomy procedures (biomaterial applied following partial or total amputation of the dental pulp) [5]. Moreover, clinical management of immature necrotic teeth might include regenerative endodontic procedures (REPs), with calcium silicate-based cements (CSCs) placement being required in the coronal portion of the root canal [6].

Contemporary endodontics offers a variety of pulp capping agents with specific properties, advantages, and drawbacks. Novel biomaterials, namely CSCs, were introduced as alternatives to the historically used calcium hydroxide [1].

Mineral trioxide aggregate (MTA) exhibits multiple desirable properties that make it suitable for several clinical applications, including vital pulp therapy and regenerative endodontic procedures [1,6,7,8,9,10,11,12]. Although this CSC shows characteristics that identify it as the gold standard for those clinical applications, such as low solubility, biocompatibility, hard-tissue inductive and conductive activity, antibacterial properties due to calcium hydroxide release, being set in a wet environment, and the capacity to prevent bacterial leakage, MTA has some drawbacks that include potential tooth discoloration and difficult handling [1,2,3,11,12,13,14]. Due to the prolonged setting time and its setting in the presence of hydration, its application might require more than a single appointment to allow treatment completion [3,5,13].

Considering the main clinical shortcomings regarding MTA, a new calcium silicate-based cement was introduced: Biodentine (Septodont, Saint-Maur-des-Fossés Cedex, France), which has a shorter setting time (12 min, approximately) [15] and less of a discoloration effect [14]. The Biodentine powder contains tricalcium silicate, calcium carbonate, and zirconium oxide, whereas the water-based liquid formulation includes calcium chloride with a water reducing agent [1,11,16]. This endodontic repair material presents itself as a multipurpose biocompatible product with a wide range of clinical applications, comprising those of MTA. Its biological properties include antibacterial activity [17], biocompatibility, biomineralization capacity, and bioactivity [18,19], with a greater sealing ability within its physical characteristics [18,20]. Additionally, a shorter setting time due to calcium chloride addition and higher viscosity was reported when compared to classical MTA [1,16]. Biodentine was developed as a recommended dentin substitute biomaterial and presents an alternative to ProRoot MTA (Dentsply Tulsa Dental, Johnson City, TN, USA) [11,16].

It is well known that prevention of coronal microleakage is essential for the long-term success of endodontic treatment [21], as well as for vital pulp therapies and REPs [22]. In accordance, the American Association of Endodontics (AAE) “Clinical Considerations for a Regenerative Procedure” recommends a 3–4 mm layer of glass ionomer over the biomaterial [23]. However, regarding the bonding effectiveness of restorative materials on pulp capping agents, the preferable bonding strategy (self-etch or etch and rinse) and the definition of proper timing for definitive restoration remains a concern [5,12]. Moreover, few studies have reported conclusive data on the bond strength of composite resins to ProRoot MTA—and even fewer on the bond strength of composite resins to Biodentine—or the bond strength of CSCs to Fuji IX, a glass ionomer cement (GIC) used as a liner over pulp capping agents [1].

The purpose of the present in vitro study was to assess the proper time to perform restorative procedures, immediately (12 min) or seven days after placement of two CSC materials (ProRoot MTA or Biodentine), and to test the performance of two different restorative protocols (Prime & Bond Active, Dentsply Detrey GmgH, Konstanz, Germany), a resin-based flowable composite (SDR) (Dentsply Detrey GmgH, Konstanz, Germany) versus a Fuji IX glass ionomer (GC Corporation, Tokyo, Japan), regarding shear bond strength.

The tested null hypothesis stated there were no statistically significant differences between the five tested groups regarding the adhesive joints’ shear bond strength value.

## 2. Results

Pre-test failures occurred in three specimens from group MTA/GIC and were not included in the analysis (Table 1). 

There were statistically significant differences χ^2^ (4) = 23.72; *P* < 0.001 regarding the shear bond strength in the tested groups.

Concerning the 12-min groups, the Biodentine group (Biodentine/immediate SDR) showed the highest mean shear bond strength value (Table 2), with statistically significant differences (*P* < 0.05) when compared to both immediate MTA groups (MTA/GIC and MTA/immediate SDR). Regarding the 12 min MTA samples, MTA/GIC and MTA/immediate SDR did not present statistical differences in the referred parameter. Therefore, no statistically significant differences were verified between both restorative materials.

Within the 7-day groups (MTA/delayed SDR and Biodentine/delayed SDR), there were no statistical differences between both biomaterials. 

Regarding the two time intervals, the 7-day MTA group (MTA/delayed SDR) presented statistically significant differences (*P* < 0.05) compared to the 12-min MTA group when bonded to SDR (MTA/immediate SDR), whereas no differences were reported regarding Biodentine performance (Biodentine/immediate SDR and Biodentine/delayed SDR). Regarding both restorative material types and placement timing, SDR restoration 7 days after CSC application (group MTA/delayed SDR) presented a statistically significant (*P* < 0.05) higher mean bond strength value compared to GIC placed immediately (group MTA/GIC).

Concerning fracture pattern, no specimens failed cohesively within the restorative material (GIC or SDR) and no mixed failures were verified (Table 2). All MTA samples exhibited cohesive failures regardless of the restorative material placement timing. Biodentine fracture pattern analysis revealed a primarily cohesive fracture within the substrate in the 12-min group, whereas the adhesive failure mode presented a higher rate in the 7-day group.

## 3. Discussion

As there are no available guidelines in performing restorative procedures in vital pulp therapies or REPs, the purpose of the present in vitro study was to assess the most effective timing for restoration, immediately or delayed, after CSC material placement. An insight into this parameter would present undoubted clinical relevance, because if multiple visit procedures could be suitably performed in a single visit, decreasing both time and cost, early restorative time intervals could become an appropriate and evidence-based approach. 

In the present study, statistically significant differences were verified regarding the shear bond strength among the tested groups. Therefore, the null hypothesis was rejected. In fact, our results suggest that restorative procedures performed 12 min after Biodentine placement present similar bonding effectiveness to a delayed timeframe. In contrast, restoration timing following MTA procedures might present a key factor to success, since a delayed timing was associated with a statistically significant increase in the adhesion values. These findings might reflect the demand for multiple visits for MTA, while Biodentine allows for immediate restoration. 

While Biodentine exhibited a 12-min setting time, MTA presented a prolonged setting time of 2 h and 45 min [8,12,24]. Few researchers have evaluated the shear bond strength of MTA or Biodentine to resin-based composites at different time intervals [8,12,13,16,25]. Neelakantan et al. [8] assessed the bond strength between resin-based composites and MTA, applying three different bonding systems. This study’s results were by far the highest found within the literature, presenting in some cases mean shear bond strength (SBS) values close to dentin adhesion values with no methodology traits justifying such a difference [8]. Tsujimoto et al. [12] and Hashem et al. [25] presented opposing results to those found in our study, stating that almost immediate placement of the final restoration over MTA during a single appointment, and delayed restoration (at least two weeks) over Biodentine, are preferable. On the contrary, Atabek at al. [26] showed that higher shear bond strength measurements of MTA could be obtained at 72 h, and that after this period the SBS values did not change dramatically. Concerning Biodentine, and aligned with our results, Meraji and Camilleri [27] reported a mean SBS value of approximately 4 MPa in restorations using an etch and rinse technique over Biodentine, and they also reported a total loss of composite resins bonded with a self-etch adhesive system following thermocycling. In our study, SDR was bonded to both MTA and Biodentine, applying a universal adhesive system. Previous acid etching was not performed considering surface treatment effects remain unclear within the literature and several authors have described MTA surface degradation, cohesive strength reduction, modified microhardness, affected compressive strength, and displacement and dissolution of MTA, with amorphous surface structures and needle-shaped crystal removal following acid etching [5,22,28]. Tsujimoto et al. [12] performed a self-etching procedure and reported no MTA microhardness disturbance in a 10-min group. 

Regarding both restorative material and placement timing, SDR restoration 7 days after CSC application (group MTA/delayed SDR) presented a statistically significant (*P* < 0.05) higher mean bond strength value compared to GIC placed immediately (group MTA/GIC), which might be related to the underlying adhesive mechanisms. These findings suggest that restorative procedures might be preferably performed in a delayed timeframe. Moreover, although groups MTA/GIC and MTA/immediate SDR presented no statistical differences, it is noteworthy that four GIC specimens presented fracture during handling. Regarding GIC retention, both chemical and micromechanical adhesion were suggested as mechanisms for bonding to a calcium silicate-based cement [8]. Apart from these findings, as there are no literature references whatsoever on this topic, it is important to clarify in further studies if the 10-MDP present in universal adhesives would also chemically interact via ionic bonding to calcium present in calcium silicate-based cement as it does to calcium present in hydroxyapatites [29]. It is worth mentioning that the tested adhesive area was defined based on previous studies’ methodologies [1,5,9,12,30]. However, considering the results of the present study regarding the high frequency of observation of cohesive fracture patterns within MTA (100%, regardless of the restoration timing) and Biodentine (approximately 50%, regardless of the restoration timing), it may be advisable to perform testing on smaller adhesive areas in further studies when assessing CSC.

Concerning fracture patterns, in our study no specimens failed cohesively within the composite resin, similarly to the findings of Odabas et al. [16]. When comparing both calcium silicate-based cement groups (MTA vs. Biodentine), regardless of the restorative material or timing, the differences in fracture pattern may be due to a different intrinsic cohesive strength between MTA and Biodentine, the latter probably presenting better physical and mechanical properties. Failure mode analysis highlighting a cohesive fracture pattern within pulp capping material might reflect its low cohesive resistance compared to a high bond strength value [22,30]. In fact, in the present study, all MTA samples exhibited cohesive failures regardless of the restorative material placement timing, therefore suggesting a favorable bond between MTA and both tested restorative materials. 

In addition, as previously mentioned, there was a significant difference between the mean bond strengths at 12 min and 7 days for the MTA groups bonded to SDR, with the highest value obtained in the delayed timing group. Therefore, considering all failures were cohesive in the calcium silicate-based cement and a statistically higher bond strength value was obtained at 7 days, MTA might present a superior cohesive resistance 7 days after placement. Furthermore, previous studies analyzed the interface obtained when Fuji IX was placed over unset MTA [31]. Findings showed that GICs were responsible for water sorption from the freshly mixed hydraulic calcium silicate-based cement, leading to a higher porosity and incomplete hydration of MTA [31]. The interface region between GIC and MTA exhibited a high degree of microcracking, with the two materials pulling away from each other, leaving some of the MTA attached to the glass ionomer cement [31]. These findings present a possible explanation for the MTA/GIC specimen results in the present study concerning the pre-test fractures, the lowest mean shear bond strength value, and the exclusively cohesive fracture pattern within MTA. Concerning Biodentine failure pattern analysis, a primarily cohesive fracture within the subtract in the 12-min group was recorded, whereas the adhesive failure mode presented a higher rate in the 7-day group. Furthermore, considering Biodentine samples showed no bond strength differences when both time intervals were compared, a slightly increased number of adhesive failures within the pulp capping material in the delayed group might suggest a lower bond strength level or a higher biomaterial cohesive strength. 

Another concern related to this study is the discoloration potential associated with both ProRoot MTA and Biodentine [32]. Ramos et al. [14] showed that MTA induces higher discoloration after six weeks of treatment, and the discoloration increased over time up to one year of clinical performance, whereas teeth treated with Biodentine presented better color stability.

## 4. Materials and Methods

### 4.1. Sample Preparation and Restorative Material Placement

A total of 75 acrylic blocks (3 cm in height × 1.5 cm in diameter) presenting with a 5 mm diameter and a 2 mm deep central hole (standard position and dimensions obtained using a putty (Virtual Refill Putty Fast Set, Ivoclar Vivadent AG, Schaan, Liechtenstein) mold) were prepared and polished. The blocks were then randomly divided into 5 groups (*n* = 15) for the analysis (Table 2). 

### 4.2. Groups 1, 2, and 3 (ProRoot MTA)

**Figure 1:**
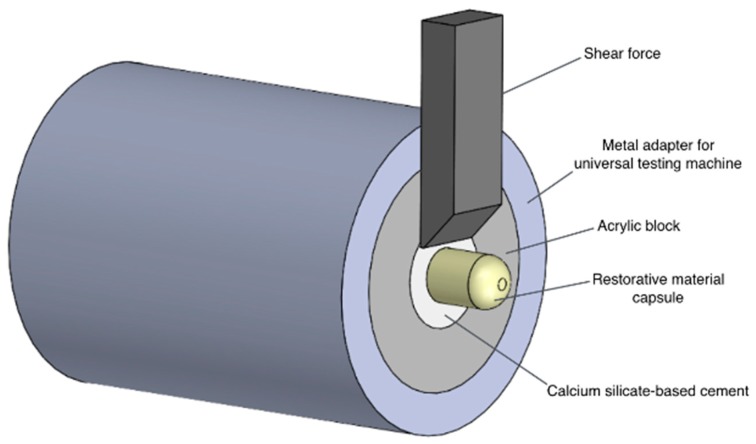
Schematic illustration of sample design and positioning during shear bond strength test.

All specimens were filled with ProRoot MTA (Figure 1). The CSC was prepared according to the manufacturer’s instructions (described in Supplementary Materials—Table S1), was applied to the acrylic blocks using a spatula, and was compacted with a vertical plugger (Buchanan Hand Plugger #2, SybronEndo, Orange, CA, USA). 

Regarding group 1 (MTA/GIC using AAE standard technique), no adhesive resin was used. In group 2 (MTA/immediate SDR), 12 min after biomaterial placement, application of a universal bonding system (Prime & Bond Active) over the capping material was performed, without acid etching. Subsequently, a cylindrically shaped capsule with an internal diameter of 2.54 mm and 4.39 mm in height was filled with either Fuji IX (MTA/GIC group) or Surefil SDR bulk fill flowable composite (MTA/immediate SDR group). GIC powder and liquid were mixed according to the manufacturer’s instructions and were centrally placed over the restoration site. The SDR flowable composite was light-cured for 80 s with a polywave LED curing light source (Bluephase^®^ Style, Ivoclar Vivadent AG, Schaan, Liechtenstein) for 20 s per quadrant. Samples from the MTA/GIC and MTA/immediate SDR groups were stored in an incubator (Gallenkamp, London, UK) at 100% relative humidity and 37 °C for 48 h to allow complete setting before shear bond strength testing.

Concerning group 3 (MTA/delayed SDR), all 15 samples were stored in an incubator at 100% relative humidity and 37 °C for 7 days to allow complete setting of the CSC. After the 7-day scheduled storage, the biomaterial surface was air-dried and bonding procedures were performed following the restorative protocol described for the MTA/immediate SDR group.

### 4.3. Groups 4 and 5 (Biodentine)

For groups 4 (Biodentine/immediate SDR) and 5 (Biodentine/delayed SDR), Biodentine was prepared and applied to the acrylic blocks as previously described (Figure 1).

In the Biodentine/immediate SDR group, 12 min after CSC placement, a universal bonding system was applied over the capping material according to the manufacturer’s instructions, without acid etching. Restorative procedures were performed using SDR, as previously mentioned. Samples were stored in an incubator at 100% relative humidity and 37 °C for 48 h to allow complete setting before testing.

Regarding the Biodentine/delayed SDR group, after 7 days of storage in an incubator at 100% relative humidity and 37 °C, bonding and restorative procedures were performed similarly to the MTA/immediate SDR, MTA/delayed SDR, and Biodentine/immediate SDR groups.

### 4.4. Shear Bond Strength Test

A group’s testing sequence was randomly defined. Samples were tested in a shear mode using a universal testing machine (Model AG-I, Shimadzu Corporation, Kyoto, Japan). The compression load resulting in the shear bond strength was performed parallel and as close as possible to the adhesive interface. The shear force was applied by a chisel-shaped rod at a crosshead speed of 0.5 mm/min up to bond disruption. Shear bond strength values, expressed in MPa, were calculated by dividing peak break force (*N*) by the cross-sectional area of the bonded interface of each group.

### 4.5. Fracture Analysis

Following the SBS test, the fractured surfaces were evaluated by a single-blind operator using a stereo microscope (Nikon SMZ1500—objective HR Plan Apo 1X WD 54, Tokyo, Japan), and the fracture pattern was registered. Fracture mode was classified as follows: (0) adhesive fracture, (1) cohesive fracture in calcium silicate-based material, (2) cohesive fracture in the restorative material, and (3) mixed failure (comprising both adhesive and cohesive fracture). 

### 4.6. Statistical Analysis

The normality of data distribution testing was carried out using the Shapiro–Wilk test. Kruskal–Wallis tests were used to detect significant differences between the medians across the groups as data did not follow the normal distribution. Post-hoc comparisons were accomplished using the Dunn–Sidak test. Statistical analysis was performed using the commercially available IBM SPSS v.24 software (Chicago, IL, USA). The outcomes regarding shear bond strength were expressed in MPa.

## 5. Conclusions

Within the limitations of the present in vitro study, our findings suggest that bonding procedures directly on top of ProRoot MTA might be preferably performed at a delayed timeframe, while Biodentine might allow for immediate restoration. For this reason, the use of Biodentine can be a material of choice for immediate restoration instead of using MTA.

## Figures and Tables

**Table 1:** Mean bond strength values and fracture pattern of the tested groups following shear bond strength test.

		Restoration Timing		Fracture Pattern			
Bioceramic/Restorative material	Bonding system	Immediate (12 min)	Delayed (7 days)	Immediate (12 min)		Delayed (7 days)	
				Cohesive in bioceramic	Adhesive	Cohesive in bioceramic	Adhesive
ProRoot MTA^®^/GC Fugi IX GP	-	Group 1 *1.14 ± 1.12^b^	-	12	-	-	-
ProRoot MTA^®^/SDR^TM^	Prime & Bond Active^TM^	Group 2 *1.33 ± 1.56^b^	Group 3 *3.86 ± 1.72^a^	15	-	15	-
Biodentine^TM^/SDR^TM^	Prime & Bond Active^TM^	Group 4 *4.44 ± 2.49^a^	Group 5 *3.09 ± 2.23^a,b^	8	7	7	8

* Mean bond strength value ± standard deviation (MPa); *P*-values sharing the same superscript letter did not have a statistically significant difference (*P* > 0.05).

**Table 2:** Description of study groups, with biomaterials, restoration timing, bonding systems, and restorative materials.

Group	Calcium Silicate-Based Cement	Restoration Timing (after Bioceramic Application)	Bonding System	Restorative Material
1	ProRoot MTA	12 min	-	GC Fugi IX GP
2			Prime & Bond Active	SDR
3		7 days	Prime & Bond Active	SDR
4	Biodentine	12 min	Prime & Bond Active	SDR
5		7 days	Prime & Bond Active	SDR

## Supplementary Materials

The following are available online at http://www.mdpi.com/1996-1944/11/11/2216/s1, Table S1: Materials’ manufacturers, compositions, application procedures, lot numbers and expiration date information.
