# Supplementary material for: Does Delayed Restoration Improve Shear Bond Strength of Different Restorative Protocols to Calcium Silicate-Based Cements?
Source: Materials (Basel). 2018 Nov 8;11(11):2216. doi: 10.3390/ma11112216 (PMC6265959; doi:10.3390/ma11112216)
Supplement: Supplementary file 1 [file materials-11-02216-s001.pdf]

materials

Supplementary Material: Does Delayed Restoration Improve Shear Bond Strength of Different Restorative Protocols to Calcium Silicate-Based Cements?

Paulo J. Palma, Joana Marques, Rui I. Falacho, Alexandra Vinagre, João M. Santos and João C. Ramos

Table S1. Materials' manufacturers, compositions, application procedures, lot numbers and expiration date information.

Material	Manufacturer	Composition	Preparation/ Application Procedure	Lot Number	Expiration Date
Biodentine™ bioactive dentine substitute	Septodont, Saint-Maur-des-Fossés Cedex, France	Powder: Tricalcium silicate, zirconium oxide, calcium oxide, calcium carbonate, and colourings. Aqueous solution: Calcium chloride and polycarboxylate.	1. Pour 5 drops of liquid into the capsule 2. Place the capsule on a mixing device 3. Mix for 30 seconds	B19588	01-2019
ProRoot MTA® (White) mineral trioxide aggregate	Dentsply Tulsa Dental, Johnson City, TN, USA	Tricalcium silicate, bismuth oxide, dicalcium silicate, tricalcium aluminate, and calcium sulfate dehydrate or gypsum	Mixed powder/liquid ratio 1:3	121780	02-2018
Prime & Bond Active™ universal adhesive system	Dentsply DeTrey GmbH, Konstanz, Germany	Phosphoric acid modified acrylate resin, multifunctional acrylate, bifunctional acrylate, acidic acrylate, isopropanol, water, initiator, stabilizer	1. Apply the bonding system to all surfaces 2. Rest for 20 seconds 3. Air dry for at least 5 seconds to promote solvent evaporation 4. Light-cure for 10 seconds	1703000402	01-2019
SDR™ flowable composite	Dentsply DeTrey GmbH, Konstanz, Germany	Barium-alumino-fluoro-borosilicate glass, strontium-alumino-fluoro-silicate glass, modified urethane dimethacrylate resin, EBPADMA, TEGDMA, CQ, photoaccelerator, BHT, UV stabilizer, titanium dioxide, iron oxide pigments, fluorescing agent	1. Dispense SDR™ material 2. Light-cure for at least 20 seconds	02023	02-2020

GC Fuji IX GP (A3) glass ionomer restorative cement	GC Corporation, Tokyo, Japan	Powder: Aluminofluorosilicate glass, polyacrylic acid powder. Liquid: Polyacrylic acid, distilled water, polybasic carboxylic acid.	Mixed powder/liquid ratio 1:1	1610031	10-2019

© 2018 by the authors. Licensee MDPI, Basel, Switzerland. This article is an open access article distributed under the terms and conditions of the Creative Commons Attribution (CC BY) license (http://creativecommons.org/licenses/by/4.0/).
